# Supplementary material for: Multidimensional Phylogenetic Metrics Identify Class I Aminoacyl-tRNA Synthetase Evolutionary Mosaicity and Inter-Modular Coupling
Source: Int J Mol Sci. 2022 Jan 28;23(3):1520. doi: 10.3390/ijms23031520 (PMC8835825; doi:10.3390/ijms23031520)
Supplement: Supplementary file 1 [file ijms-23-01520-s001.zip › ijms-1539462-supplementary.pdf]

International Journal of *Molecular Sciences*

MDPI

Amino acid sequences of the two LeuAC constructs used are:

LeuAC_1:

```
GAG AAA AAA TTC TAC ATC ACC GTG GCG TTC CCG TAC ACG AGC GGC CAT CTG CAC GTT GGT CAC GCG ATT
 E   K   K   F   Y   I   T   V   A   F   P   Y   T   S   G   H   L   H   V   G   H   A   I
ACC TAT ACG ATC CCG GAT ATT ATC GCC CGT TTT AAA CGC ATG CAG GGC TAC AAT GTG CTG TTC CCG ATG
 T   Y   T   I   P   D   I   I   A   R   F   K   R   M   Q   G   Y   N   V   L   F   P   M
GCC CTG CAT ACC GAT GGT CTG ACC GAT AGT ACG ATT TAT ATG GCA GTT CTG CTG ATC CTG TAT TGG TAC
 A   L   H   T   D   G   L   T   D   S   T   I   Y   M   A   V   L   L   I   L   Y   W   Y
CCG CTG GAT TGG CGT TGC AGC GGC AAA GAT CTG ATT CCG AAC CAT CTG ACC TTT TTC ATC ATC AAC CAC
 P   L   D   W   R   C   S   G   K   D   L   I   P   N   H   L   T   F   F   I   I   N   H
GTG GCA ATC TTC CGC GAA GAA CAT TGG CCG AAA GGT ATC GCG GTT AAC GGC TTC GGT ACG CTG GAA GGC
 V   A   I   F   R   E   E   H   W   P   K   G   I   A   V   N   G   F   G   T   L   E   G
CAG AAA ATG AGC AAA TCT AAG GGT AAC GTG CTG AAT CGT ATC
 Q   K   M   S   K   S   K   G   N   V   L   N   R   I
```

LeuAC_2

```
GAA AAG AAA TTT TAT ATC ACC GTG GCC TTT CCG TAT CTG AGT GGC CAT CTG CAT GTT GGT CAT GCC CGC
 E   K   K   F   Y   I   T   V   A   F   P   Y   L   S   G   H   L   H   V   G   H   A   R
ACC TAT ACC ATT CCG GAT GAA ATT GCA CGC ACC AAA CGT AAA CAG GGC TAT AAT GTT CTG TTT CCG ATG
 T   Y   T   I   P   D   E   I   A   R   T   K   R   K   Q   G   Y   N   V   L   F   P   M
GAT TGG CAT ACC ACC AGC CTG AGC GAT AGC ACC ATC TAT ATG GCA GAA TAT ACC AGT GAA TAT TGG TAT
 D   W   H   T   T   S   L   S   D   S   T   I   Y   M   A   E   Y   T   S   E   Y   W   Y
CCG CTG GAT TGG CGC TGC AGC GGC AAA GAT CTG ATT CCG AAT CAT CTG ACC AAA TTC ATT TTT AAT CAC
 P   L   D   W   R   C   S   G   K   D   L   I   P   N   H   L   T   K   F   I   F   N   H
GTG GCA ATT TTC CGT GAA GAA CAT TGG CCG AAA GGT ATT GCC GTT AAT GGC AGT GGT ACA CTG GAA GGC
 V   A   I   F   R   E   E   H   W   P   K   G   I   A   V   N   G   S   G   T   L   E   G
CAG AAA ATG AGT AAA AGC AAA GGT AAT GTT CTG AAT TTC AGC
 Q   K   M   S   K   S   K   G   N   V   L   N   F   S
```

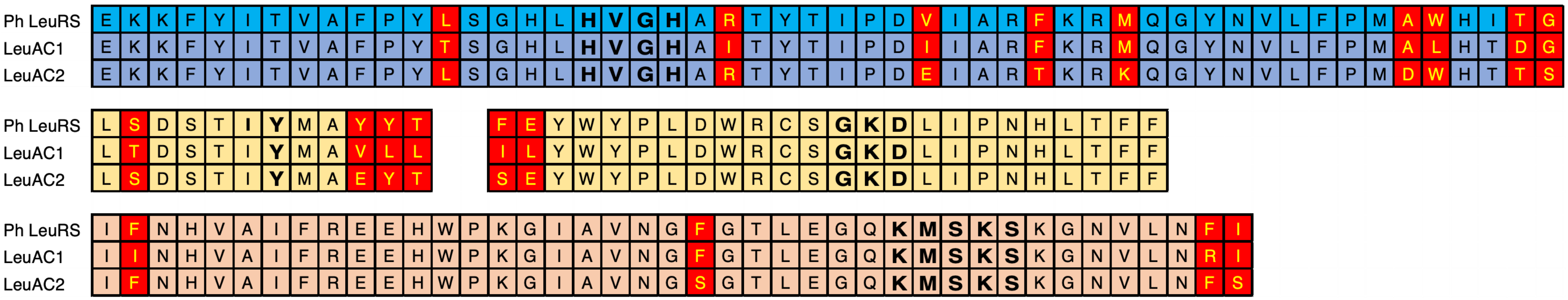

Figure S1. Sequence differences between LeuAC constructs and homologous sequence from *P. horikoshii* LeuRS. The sequence is broken into three fragments analogous to the three described by Pham, et al., (1). Connecting peptide 1 (CP1) connects the C-terminus of the blue fragment to the N-terminus of the Amber fragment. Connecting Peptide 2 (CP2) is indicated by the gap in the Amber fragment. Differences between the three sequences are highlighted in red boxes. Active site residues are highlighted in bold and larger fontsize. Entries for LeuAC2 enhance the solubility of LeuAC2, but have no detectable effect on the active site titration timecourses.

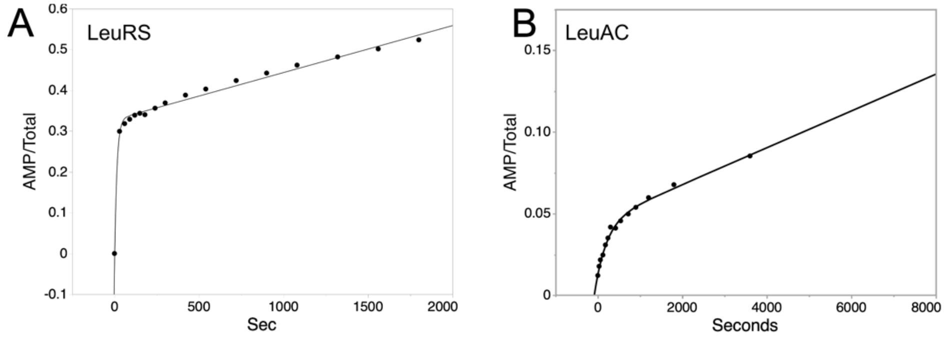

Parameter	β	StdErr	β/σ
C	0.32	0.00375	86.5
A	-0.32	0.01003	-32.3
kchem	8.1E-02	0.012	7.0
kcat	9.0E-05	4.40E-06	20.4
kchem/kcat	902		

Parameter	β	StdErr	β/σ
C	0.05	0.00215	21.1
A	-0.03	0.0022	-14.6
kchem	3.61E-03	0.00056	6.5
kcat	1.13E-5	9.51E-07	1.19E+01
kchem/kcat	319		

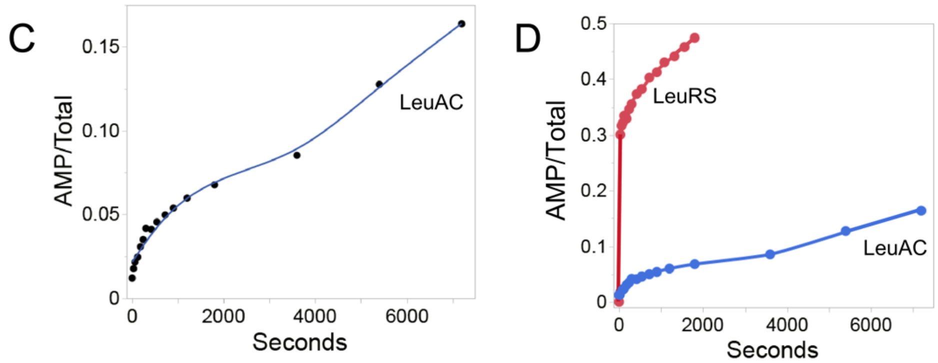

Figure S2. Timecourses for AMP formation by LeuRS and LeuAC. A. Active-site titration plot for LeuRS. B. Active-site titration plot for LeuAC, using only timepoints to 4000 seconds. C. Extended timecourse for LeuAC, showing an increased linear turnover rate from 4000 to 7200 seconds. D. LeuRS and LeuAC timecourses compared on the same coordinate system.

Table S1. Biphasic (single turnover) fits ^{32}P-ATP loss.

Sample	n	Date	C	A	kchem	kcat	kchem/kcat	ΔGkchem	ΔGkcat	ΔGkchem/kcat	URZ	TEV	α labeled	LSA	AMPcPP	*P. horik*
Ph LeuRS	1.2	6/2/21	0.68	0.32	0.08085	8.97E-05	902	1.49	5.52	-4.03	0	0	0	0	0	1
Ph LeuRS	1.3	5/25/21	0.63	0.37	0.06870	1.59E-04	431	1.59	5.18	-3.59	0	0	0	0	0	1
Ec LeuRS	1.0	12/14/20	0.13	0.29	0.00178	7.06E-06	252	3.75	7.02	-3.27	0	0	0	0	0	0
Ec LeuRS	1.6	12/14/20	0.13	0.44	0.00052	4.30E-06	120	4.48	7.32	-2.83	0	0	1	0	1	0
Ec LeuRS	1.4	12/14/20	0.29	0.39	0.00082	4.42E-06	185	4.21	7.30	-3.09	0	0	1	1	0	0
Ec LeuRS	0.9	12/14/20	0.12	0.24	0.00236	6.05E-06	389	3.58	7.11	-3.53	0	0	1	0	0	0
Ec LeuRS	1.0	12/14/20	0.13	0.29	0.00178	7.06E-06	252	3.75	7.02	-3.27	0	0	1	0	0	0
LeuAC (MBP)	2.4	8/9/18	0.26	0.68	0.00043	2.18E-05	20	4.59	6.35	-1.76	1	0	0	0	0	1
LeuAC (MBP)	2.4	5/25/21	0.26	0.68	0.00043	2.18E-05	20	4.59	6.35	-1.76	1	0	0	0	0	1
LeuAC (MBP)	2.2	12/14/20	0.43	0.62	0.00051	1.19E-05	43	4.49	6.71	-2.22	1	0	1	0	1	1
LeuAC (MBP)	2.5	12/14/20	0.37	0.69	0.00036	4.43E-06	82	4.69	7.30	-2.61	1	0	1	0	0	1
LeuAC (MBP)	2.0	12/4/20	0.42	0.57	0.00033	4.36E-06	75	4.75	7.31	-2.55	1	0	1	0	0	1
LeuAC (MBP)	2.3	12/18/20	0.35	0.65	0.00040	4.27E-06	93	4.64	7.32	-2.68	1	0	1	0	0	1
LeuAC (MBP)	2.5	12/18/20	0.31	0.69	0.00036	*ND*	*ND*	4.69	*ND*	*ND*	1	0	1	0	0	1
LeuAC (MBP)	2.4	12/18/20	0.34	0.67	0.00038	*ND*	*ND*	4.67	*ND*	*ND*	1	0	1	0	0	1
LeuAC (MBP)	2.4	12/18/20	0.33	0.67	0.00037	*ND*	*ND*	4.67	*ND*	*ND*	1	0	1	0	0	1
LeuAC (MBP)	2.3	12/18/20	0.37	0.64	0.00041	*ND*	*ND*	4.62	*ND*	*ND*	1	0	1	0	0	1
LeuAC (MBP)	2.3	12/18/20	0.35	0.65	0.00042	*ND*	*ND*	4.60	*ND*	*ND*	1	0	1	0	0	1
LeuAC (MBP)	2.4	12/18/20	0.34	0.66	0.00037	*ND*	*ND*	4.68	*ND*	*ND*	1	0	1	0	0	1
LeuAC (TEV)	1.9	5/25/21	0.41	0.52	0.00123	2.33E-05	53	3.97	6.31	-2.35	1	1	0	0	0	1
LeuAC (TEV)	2.1	5/4/21	0.27	0.60	0.00326	2.00E-05	161	3.39	6.41	-3.01	1	1	1	0	0	1
LeuAC (TEV)	2.2	5/4/21	0.26	0.62	0.00321	2.07E-05	155	3.40	6.38	-2.99	1	1	1	0	0	1
LeuAC (TEV)	2.2	10/19/17	0.42	0.61	0.00070	1.30E-05	52	4.30	6.66	-2.34	1	1	0	0	0	1
LeuAC (TEV)	2.1	10/19/17	0.43	0.59	0.00066	1.80E-05	37	4.34	6.47	-2.14	1	1	0	0	0	1
LeuAC (TEV)	2.1	5/4/21	0.27	0.58	0.00331	1.97E-05	168	3.38	6.41	-3.03	1	1	0	0	0	1

LeuAC (TEV)	1.9	8/9/18	0.41	0.52	0.00123	2.33E-05	53	3.97	6.31	-2.35	1	1	0	0	0	1

ND denotes kcat values that were determined with β/σ <1.0

Table S2. Biphasic (single turnover) fits to aminoacylation experiments.

Sample	Date	C	A	kchem	kcat	kchem/kcat	ΔGkchem	ΔGkcat	ΔGkchem/kcat	URZ	TEV	[tRNA], M	[enzyme], M	tRNA/E	14CLeu
Ph LeuRS	9/30/20	0.012	0.90	0.0083	1.77E-05	466	2.84	6.48	-3.64	0	0	5.00E-06	5.00E-07	10.0	0
Ph LeuRS	9/24/20	0.001	0.97	0.0026	3.10E-05	83	3.54	6.15	-2.61	0	0	1.00E-05	5.00E-06	2.0	0
Ph LeuRS	9/24/20	0.291	0.68	0.0036	1.53E-04	23	3.34	5.20	-1.87	0	0	1.00E-05	5.00E-06	2.0	0
Ph LeuRS	10/5/20	0.355	0.32	0.0100	3.80E-04	27	2.72	4.66	-1.95	0	0	5.33E-06	5.00E-06	1.1	0
Ph LeuRS	11/23/20	0.084	0.93	0.0046	3.51E-05	130	3.19	6.07	-2.88	0	0	3.24E-05	5.00E-06	6.5	1
Ph LeuRS	11/17/20	0.694	0.31	0.0181	3.19E-04	57	2.38	4.77	-2.39	0	0	1.60E-05	2.60E-05	0.6	1
Ph LeuRS	10/7/20	0.056	0.94	0.0031	3.96E-05	77	3.43	6.00	-2.58	0	0	5.00E-06	1.25E-05	0.4	0
Ph LeuRS	10/7/20	0.137	0.86	0.0041	7.18E-05	57	3.26	5.65	-2.39	0	0	5.00E-06	1.25E-05	0.4	0
Ph LeuRS	10/7/20	0.092	1.15	0.0051	6.68E-05	77	3.13	5.69	-2.57	0	0	4.30E-05	1.25E-05	3.4	0
Ph LeuRS	10/7/20	0.278	0.49	0.0046	1.45E-04	31	3.19	5.23	-2.04	0	0	4.30E-05	1.25E-05	3.4	0
Ph LeuRS	10/7/20	0.356	0.53	0.0041	1.85E-04	22	3.26	5.09	-1.83	0	0	4.30E-05	1.25E-05	3.4	0
Ph LeuRS	10/28/20	0.096	0.90	0.0538	1.97E-04	274	1.73	5.05	-3.32	0	0	2.60E-05	1.30E-05	2.0	1
Ph LeuRS	10/28/20	0.201	0.80	0.0819	2.01E-04	407	1.48	5.04	-3.56	0	0	2.60E-05	1.30E-05	2.0	0
Ph LeuRS	10/9/20	0.292	0.70	0.0440	2.43E-04	179	1.86	4.93	-3.07	0	0	4.60E-08	1.25E-05	0.0	0
Ph LeuRS	10/9/20	0.384	0.60	0.0240	1.84E-04	128	2.22	5.09	-2.87	0	0	1.67E-07	1.25E-05	0.0	0
Ph LeuRS	10/9/20	0.044	0.91	0.0150	7.00E-05	219	2.48	5.67	-3.19	0	0	2.80E-06	1.25E-05	0.2	0
Ph LeuRS	10/6/20	0.016	1.01	0.0092	5.28E-06	1744	2.78	7.19	-4.42	0	0	5.33E-06	5.00E-06	1.1	0
Ph LeuRS	10/6/20	-0.021	0.90	0.0053	6.32E-06	831	3.11	7.09	-3.98	0	0	5.33E-06	5.00E-06	1.1	0
Ph LeuRS	10/6/20	0.180	0.61	0.0063	9.82E-05	64	3.01	5.46	-2.46	0	0	5.33E-06	5.00E-06	1.1	0
Ph LeuRS	10/6/20	-0.153	0.75	0.0053	6.00E-05	88	3.11	5.75	-2.65	0	0	5.33E-06	5.00E-06	1.1	0
LeuAC (MBP)	9/4/19	0.379	0.55	0.0007	9.00E-05	8	4.32	5.52	-1.20	1	0	6.00E-05	5.00E-06	12.0	0
LeuAC (MBP)	3/12/21	0.191	0.81	0.0010	5.48E-05	18	4.11	5.81	-1.70	1	0	9.13E-05	9.10E-06	10.0	0
LeuAC (MBP)	3/24/19	0.230	0.60	0.0029	2.50E-05	116	3.46	6.27	-2.82	1	0	6.00E-05	5.00E-06	12.0	0

LeuAC (MBP)	4/26/21	0.157	0.85	0.0023	5.91E-05	38	3.61	5.76	-2.16	1	0	8.02E-05	3.60E-05	2.2	0
LeuAC (TEV)	3/12/21	-0.017	1.02	0.0021	5.44E-06	387	3.65	7.18	-3.53	1	1	9.13E-05	5.40E-06	16.9	0
LeuAC (TEV)	2/25/19	0.414	0.34	0.0039	3.00E-04	14	3.28	4.84	-1.56	1	1	1.50E-05	5.00E-06	3.0	0
LeuAC (TEV)	3/29/19	0.526	0.47	0.0270	2.48E-04	108	2.15	4.92	-2.77	1	1	5.00E-06	5.00E-06	1.0	0
LeuAC (TEV)	3/22/19	0.258	0.74	0.0491	8.47E-04	58	1.79	4.19	-2.40	1	1	6.00E-05	5.00E-06	12.0	0

References

1. Pham, Y., Li, L., Kim, A., Erdogan, O., Weinreb, V., Butterfoss, G., Kuhlman, B. and Carter, C.W., Jr. (2007) A Minimal TrpRS Catalytic Domain Supports Sense/Antisense Ancestry of Class I and II Aminoacyl-tRNA Synthetases. *Mol Cell*, **25**, 851-862.
